# Transparent, Conductive Hydrogels with High Mechanical Strength and Toughness

**DOI:** 10.3390/polym13122004

**Published:** 2021-06-18

**Authors:** Xiuru Xu, Chubin He, Feng Luo, Hao Wang, Zhengchun Peng

**Affiliations:** 1 Guangdong Provincial Key Laboratory of Micro/Nano Optomechatronic Engineering, College of Mechatronics and Control Engineering, Shenzhen University, Shenzhen 518060, China; xiuruxu@foxmail.com (X.X.); LLF@szu.edu.cn (F.L.); whao@szu.edu.cn (H.W.); 2 School of Physics and Optoelectronic Engineering, Shenzhen University, Shenzhen 518060, China; hcbfighting@163.com

**Keywords:** transparent hydrogels, toughness, wearable sensors

## Abstract

Transparent, conductive hydrogels with good mechanical strength and toughness are in great demand of the fields of biomedical and future wearable smart electronics. We reported a carboxymethyl chitosan (CMCS)–calcium chloride (CaCl_2_)/polyacrylamide (PAAm)/poly(N-methylol acrylamide (PNMA) transparent, tough and conductive hydrogel containing a bi-physical crosslinking network through in situ free radical polymerization. It showed excellent light transmittance (>90%), excellent toughness (10.72 MJ/m^3^), good tensile strength (at break, 2.65 MPa), breaking strain (707%), and high elastic modulus (0.30 MPa). The strain sensing performance is found with high sensitivity (maximum gauge factor 9.18, 0.5% detection limit), wide strain response range, fast response and recovery time, nearly zero hysteresis and good repeatability. This study extends the transparent, tough, conductive hydrogels to provide body-surface wearable devices that can accurately and repeatedly monitor the movement of body joints, including the movements of wrists, elbows and knee joints. This study provided a broad development potential for tough, transparent and conductive hydrogels as body-surface intelligent health monitoring systems and implantable soft electronics.

## 1. Introduction

Due to their softness, wetness, biocompatibility, permeability, conductivity etc., 3D-network structure conductive hydrogels have attracted extensive interest [1,2]. Therefore, researchers have explored many promising applications of hydrogels with unique structures and properties, including wound healing [3], extracellular matrix [4], tissue engineering [5], implantable neural electrodes [6,7,8,9], wearable electronics [10,11] and other aspects [12,13,14,15,16]. Transparency is also an important requirement for future wearable electronics, as well as implantable electronics where see-through optical monitoring or surgical operations are needed [17,18]. Moreover, due to the mechanical properties of hydrogels, their practical application is often greatly restricted [19]. Generally, hydrogels with good stretch go with poor toughness and large hysteresis. That is because the traditional hydrogels lack an effective energy dissipation mechanism due to the uneven network structure. Much progress has been achieved in improving their mechanical properties by adding reversible sacrificial bonds to the hydrogels. For example, Peng and co-workers utilized a crosslinked hydrogel with Fe^3+^ as the ion coordination, elongation of which was about 700%, tensile strength reached 6 MPa and toughness reached 27 MJ/m^3^ [20]. Lei and co-workers reported a crosslinked hydrogel from cellulose nanocrystals to form hydrophobic forces with the polymers. Its tensile strength was about 0.3 MPa with elongation around 4000% [21]. However, either the incorporation of Fe^3+^ or cellulose nanocrystals above leads to a totally black or milky white non-transparency hydrogel. To this end, it is still challenging to design a hydrogel that combines mechanical strength and toughness with high transparency, high conductivity and long elongation.

Here, we introduced the cross-linking agent hydroxymethyl acrylamide (NMA) with an active hydroxyl group, used polyacrylamide (PAAM) as a matrix, and combined this with strong coordination of carboxymethyl chitosan (CMCS) and metal ions Ca^2+^ to successfully prepare a CMCS-Ca^2+^/PAAm/PNMA transparent conductive hydrogel (water content ~74.29%), containing a bi-physical crosslinking network with both high toughness and strength, rapid response and recovery, and good fatigue resistance. The as-prepared hydrogel utilized NMA to form the main skeleton of the hydrogel with PAAm through strong hydrogen bond interactions. Moreover, when it is subjected to external forces, there are non-covalent bonds between PAAm, CMCS and Ca^2+^, which effectively act as sacrificial bonds. Transient non-covalent bonds cross-linking networks were formed inside the hydrogel through the hydrogen bonds between CMCS and PAAm molecules, as well as the metal-ligand coordination bonds between Ca^2+^ and CMCS, thereby improving the density and mechanical properties of the hydrogel crosslinked network.

Owing to the existence of the dual physical cross-linking network, CMCS-Ca^2+/^PAAm/PNMA conductive hydrogel showed good resilience and fatigue resistance. Moreover, the conductive hydrogel showed good strain sensing performance with high sensitivity (Maximum GF = 9.1802, lowest detection limit was 0.5% strain), wide strain response range, fast response and recovery time, nearly zero hysteresis and high repeatability. We also demonstrated the as-prepared hydrogel to monitor the movement of body joints, including the movements of wrists, elbows and knee joints. This study provided an effective strategy for the design and manufacture of a new generation of body-surface intelligent health monitoring systems and implantable soft electronic devices.

## 2. Materials and Methods

Firstly, 5.00 g water and 1.00 g acrylamide monomer (AAm, 99.00%, Aladdin Bio-Chem Technology Co., Shanghai, China) were weighed in a clean glass bottle and stirred until the AAm was completely dissolved. Subsequently, 0.50 g of carboxymethyl chitosan (CMCS, Aladdin Bio-Chem Technology Co., Shanghai, China) was added to the AAm aqueous solution, the temperature was increased to 80 °C, and the mixture was stirred and dissolved for 3 h. Then, different masses (0.01, 0.10, 0.30 g) of calcium chloride (CaCl_2_, 99.0%, Aladdin Bio-Chem Technology Co., Shanghai, China) were added. After CaCl_2_ was dissolved, the temperature was lowered down to room temperature. Subsequently, 0.10 g of N-methylol acrylamide (NMA, 98%, Aladdin Bio-Chem Technology Co., Shanghai, China) crosslinking agent and 0.03 g of ammonium persulfate (APS, 98%, Aladdin Bio-Chem Technology Co., Shanghai, China) were weighed in sequence, stirred and dissolved evenly. Afterwards, it was centrifuged for 7 min (7800 rad/min) to remove air bubbles. After centrifugation, the uniform solution was poured into the template. Finally, it was allowed to stand for 2 min and cured at 50 °C for 200 min. The prepared conductive hydrogel was rinsed to wash away the unreacted monomers or other chemical substances.

The visible light transmittance of the conductive hydrogel samples was measured by using an Ultraviolet, visible and near-infrared spectrophotometer (Lambda 950, PerkinElmer, Waltham, MA, USA). The chemical structure of the conductive hydrogel samples was analyzed by a Fourier transform infrared (FT-IR) spectrometer (Nicolet 6700, Thermo Fisher Scientific, Waltham, MA, USA) with total reflection infrared spectroscopy (ATR-IR).

The tensile test was performed by the INSTRON E1000 (Instron, Norwood, MA, USA), with samples diameter of 17 mm × 6 mm × 0.3 mm, and at a tensile speed of 100 mm/min. The elastic modulus was calculated based on the slope of the linear part in the stress–strain curve of the samples. The toughness was calculated by integrating the area of the stress–strain curve.

The tensile machine (INSTRON E1000, USA) and digital multimeter (Keysight Technologies, Santa-Rosa, CA, USA) were used to test the strain sensing performance of conductive hydrogel samples. First, the hydrogel was fixed on the tensile machine. Meanwhile, the two ends of the hydrogel were connected to the digital multimeter and used the digital multimeter to record the resistance change curve of the hydrogels with strain.

## 3. Results and Discussion

### 3.1. Characterazation of CMCS-Ca^2+^/PAAm/PNMA Transparent Conductive Hydrogels

**Figure 1:**
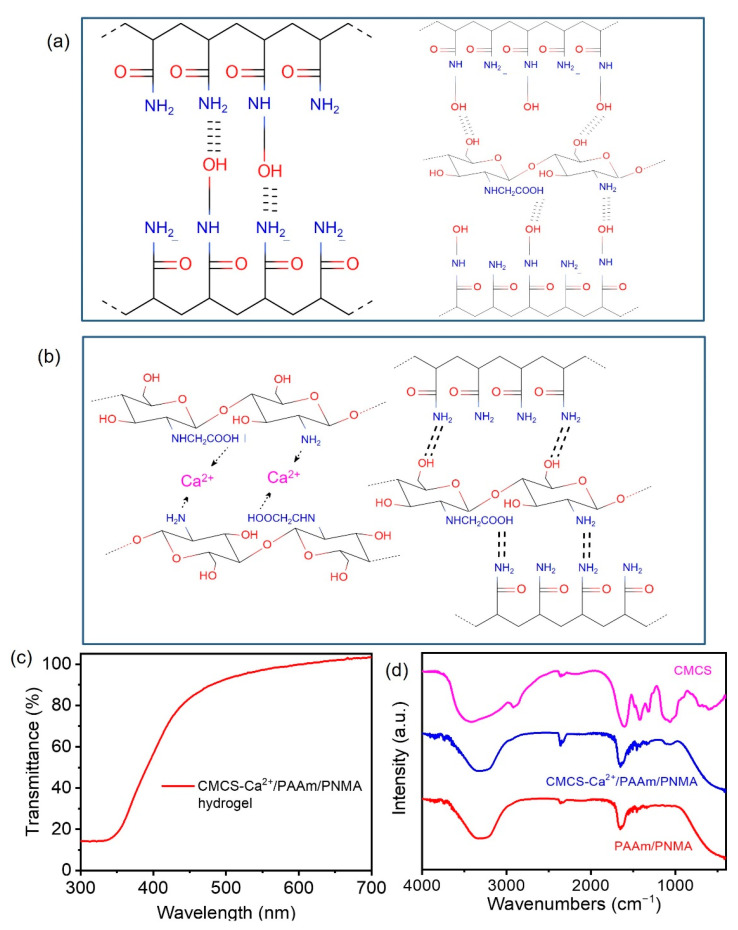
Illustration of the internal crosslinked networks of the as-prepared CMCS-Ca^2+^/PAAm/PNMA hydrogels of (**a**) strong hydrogen bond crosslinking from poly(N-methylol acrylamide) (PNMA) and (**b**) weak hydrogen bond and metal coordination interaction between carboxymethyl chitosan (CMCS) and Ca^2+^; (**c**) ultraviolet–visible (UV–vis) transmittance spectra of the as-prepared CMCS-Ca^2+^/PAAm/PNMA hydrogels; (**d**) Fourier transform infrared spectoscopy (FT-IR) spectra of CMCS, PAAm/PNMA and CMCS-Ca^2+^/PAAm/PNMA hydrogels.

Here, we used the crosslinking agent N-methylol acrylamide (NMA) with an active methylol group, which can form a strong hydrogen with the -NH_2_ group of acrylamide and the -COOH group of carboxymethyl chitosan (CMCS) (Figure 1a). In addition, CaCl_2_ was introduced into the gel as conductive ions, which can also form metal ion bonds with carboxymethyl of CMCS (Figure 1b) to increase the strength and toughness of the hydrogel. Finally, a double-network CMCS-Ca^2+^/PAAm/PNMA transparent hydrogel with strong hydrogen bonds crosslinking and Ca^2+^-COOH metal ions interaction was prepared by thermally initiating in-situ polymerization. It is worth noting that we recommend dissolving NMA at room temperature, and thermally initiated in situ polymerization at about 50 °C due to the strong reactivity of NMA.

The CMCS-Ca^2+^/PAAm/PNMA conductive hydrogel showed excellent light transmittance (Figure 1c). The light transmittance of the conductive hydrogel in the 500–700 nm wavelength range was about 90%, measured by an ultraviolet, visible and near-infrared spectrophotometer (Lambda 950, PerkinElmer, Waltham, MA, USA). Figure 1d showed the Fourier Transform Infrared Spectroscopy (FT-IR) spectra of different samples characterized by a FT-IR spectrometer (Nicolet 6700, Thermo Fisher Scientific, Waltham, MA, USA). –C=O in the amide group was found at 1649 cm^−1^. Bands at 1450 cm^−1^ and 3356 cm^−1^ are assigned to -CH_2_-CH_2_- stretching vibration peak and the –NH_2_ in the amide group stretching vibration. Moreover, the –OH hydrogen bond crosslinking with NMA and the –OH stretching vibration peak of NMA were found at 1036 cm^−1^ and 3217 cm^−1^, respectively [22,23]. These results indicated that there were hydrogen bonds in PAAm-PNMA framework, and the physical crosslinking network was formed. In addition, The N–H and C=O stretching vibration absorption peaks of carboxymethyl chitosan were found at 1601 cm^−1^ and 1062 cm^−1^ respectively [24,25]. It indicated that carboxymethyl chitosan was successfully introduced into the conductive hydrogel.

### 3.2. Mechanical Properties of CMCS-Ca^2+^/PAAm/PNMA Transparent Conductive Hydrogels

**Figure 2:**
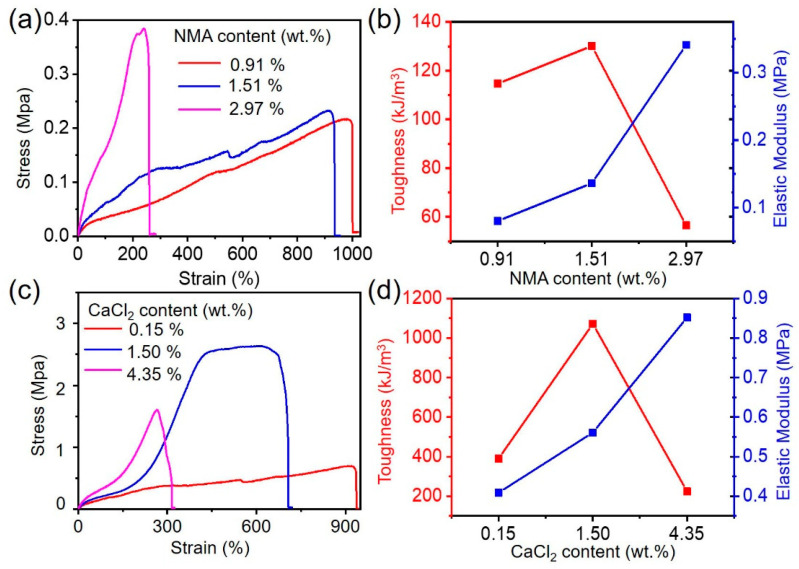
Mechanical properties of CMCS-Ca^2+^/PAAm/PNMA hydrogels. (**a**) The stress–strain behavior and (**b**) the corresponding toughness and elastic modulus of the CMCS-Ca^2+^/PAAm/PNMA hydrogels as a function of different NMA content; (**c**) the stress–strain behavior and (**d**) the corresponding toughness and elastic modulus of the CMCS-Ca^2+^/PAAm/PNMA hydrogels as a function of different addition of CaCl_2_ content.

To verify the mechanical properties of CMCS-Ca^2+^/PAAm/PNMA conductive hydrogels, we first performed uniaxial tensile experiments on hydrogels with different NMA content. It should be noted that the contents of the component mentioned here were the mass fraction of the component if there is no additional explanation. As shown in Figure 2a, as the NMA content increased, the elongation at break of the CMCS-Ca^2+^/PAAm/PNMA conductive hydrogels gradually decreased, while the tensile strength gradually increased. In addition, as the NMA content increased, the elastic modulus of the CMCS-Ca^2+^/PAAm/PNMA hydrogel increased from 0.08 MPa to 0.34 MPa, while its toughness increased from 114.7 kJ/m^3^ at 0.91 wt.% NMA to 130.2 kJ/m^3^ with 1.50 wt.% of NMA, and then decreased to 56.50 kJ/m^3^ with 2.97 wt.% of NMA (Figure 2b).

Meanwhile, we also explored the influence of different CaCl_2_ content on the mechanical properties of CMCS-Ca^2+^/PAAm/PNMA conductive hydrogels. It can be seen from Figure 2c that as the content of CaCl_2_ increased, the fracture strain of the hydrogel gradually decreased from 935% to 319%, while the tensile strength increased from 0.70 MPa to 2.65 MPa, and then decreased to 1.61 MPa. This was because too much CaCl_2_ cannot form stable metal coordination bonds with CMCS [26,27]. In addition, the toughness and elastic modulus of the hydrogel also increased first and then decreased (Figure 2d). When the CaCl_2_ content was 1.49 wt.%, the CMCS-Ca^2+^/PAAm/PNMA conductive hydrogel had the best mechanical properties with tensile strength of 2.65 MPa, breaking strain of 707%, toughness of 1071.9 kJ/m^3^ and elastic modulus of 0.30 MPa.

The excellent mechanical performance of the CMCS-Ca^2+^/PAAm/PNMA can be explained as follows: first, the introduction of NMA increased the cross-linking degree of the hydrogel network, and an effective Ca^2+^ and –COOH metal coordination bond was formed due to the addition of CaCl_2_, further improving the strength of the CMCS-Ca^2+^/PAAm/PNMA conductive hydrogel. Secondly, due to the formation of strong hydrogen bond crosslinks in the hydrogel and the Ca^2+^ and –COOH metal ions interaction, there was an excellent bi-physical crosslinking network formed, thereby effectively improving the energy dissipation of the conductive hydrogel under large strains, resulting in higher toughness.

**Figure 3:**
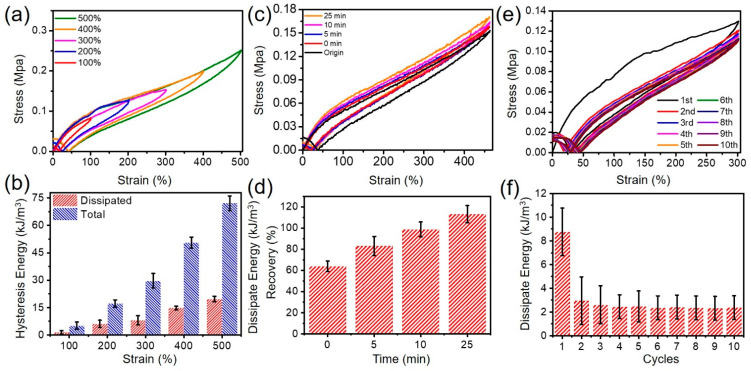
Hysteresis and self-recovery properties of CMCS-Ca^2+^/PAAm/PNMA hydrogels. (**a**) Representative stretching-relaxation tensile measurement with a series of strains (100%, 200%, 300%, 400% and 500%) and (**b**) the corresponding hysteresis energy and dissipated energy. (**c**) Cyclic tensile measurement with different rest intervals (0% to 400% strain with origin, 0 min, 5 min, 10 min and 25 min rest intervals); (**d**) the corresponding recovery ratio; (**e**) 10 stretching–relaxation cycles and (**f**) the corresponding dissipated energy.

In order to further explore the toughness enhancement mechanism of CMCS-Ca^2+^/PAAm/PNMA conductive hydrogels, we performed cyclic loading/unloading measurements on CMCS-Ca^2+^/PAAm/PNMA conductive hydrogels under different stretching strains to evaluate the energy dissipation capacity. As shown in Figure 3a,b, during the load–unload tensile measuring of the as-prepared hydrogels under different stretching strains, an obvious hysteresis circle and slight deformation after it released the load was observed, due to the overcoming of consumption work caused by frictional resistance of internal polymer segments. In addition, as the tensile strain increased from 100% to 500%, the hysteresis behaviour became more pronounced. The energy dissipated was always equal to the area of the hysteresis loop surrounded by the stretching–relaxation curves [28,29,30]. When the CMCS-Ca^2+^/PAAm/PNMA conductive hydrogel was stretched to a larger strain, it can effectively dissipate more energy and increase the mechanical strength of the hydrogel. As shown in Figure 3b, when the tensile strain was 500%, the energy dissipated by the CMCS-Ca^2+^/PAAm/PNMA hydrogel can reach 19.67 kJ/m^3^, the energy dissipation rate was 27.25%, while the energy dissipation of the hydrogel was only 1.37 kJ/m^3^, and the energy dissipation rate was 26.58% at 100% tensile strain. This phenomenon may be due to the synergy of multiple dynamic reversible non-covalent interactions among PAAm, CMCS and Ca^2+^. With the gradual increase of the tensile strain and the increase of the applied deformation, the dynamic hydrogen bonds and the metal coordination bonds as the reversible sacrifices were gradually destroyed to consume much energy. These destroyed non-covalent bonds can be reversibly linked after the external forces were removed. These results once again proved that the breaking of reversible non-covalent bonds can effectively dissipate energy, and also explained the excellent mechanical properties of CMCS-Ca^2+^/PAAm/PNMA hydrogels.

In addition, CMCS-Ca^2+^/PAAm/PNMA transparent conductive hydrogels had good self-recovery performance and fatigue resistance capacity. The hydrogel samples were subjected to cyclic stretching-relaxation cycle experiments under 500% strain at 25 °C with different relaxation time intervals (0 min, 5 min, 10 min and 25 min) to evaluate the self-recovery performance of the as-prepared CMCS-Ca^2+^/PAAm/PNMA samples. Figure 3c shows there is obvious hysteresis behaviour during the loading–unloading cycle. As the relaxation time interval increased, the hysteresis area of the hydrogel after self-recovery gradually approaches the original area of the hydrogel. Figure 3d showed that the CMCS-Ca^2+^/PAAm/PNMA hydrogel can recover 83.08% of its dissipated energy after standing for 5 min. When the resting time was 10 min, the energy dissipated recovered to 98.69%. Then the resting time increased to 25 min, and the energy dissipation returned to its original state (the energy dissipated recovered to 113.09%). As mentioned above, non-covalent bonds broke and effectively dissipated energy. These broken reversible bonds could be partially recovered within a certain relaxation time interval after the load was released and had good self-recovery properties. The longer the relaxation time interval, the more broken hydrogen bonds and coordination bonds will be restored, so the energy dissipation and self-recovery ability of the hydrogel will be greater.

In order to further evaluate the anti-fatigue performance of CMCS-Ca^2+^/PAAm/PNMA transparent conductive hydrogel, 10 cycles of load-unload tensile tests at 300% applied strain were carried out with no relaxation time between each cycle. As shown in Figure 3e,f, the tensile stress at break in the following cycles declined slightly, compared to the first cyclic tensile test. The hysteresis loop of the first cycle stretching was more obvious, and the energy dissipation was 8.8 kJ/m^3^. Starting from the second cycle, the loading–unloading cycle stretching hysteresis area of the hydrogel has been significantly reduced, which is mainly due to the fact that part of the hydrogel’s double physical cross-linking network cannot be recovered quickly after being subjected to tensile force. The cross-linked network of polymer chains became relaxed, resulting in a slight decrease in the maximum tensile stress of the hydrogel. Subsequently, the dissipation energy and tensile stress of the hydrogel stabilized. The results showed that the CMCS-Ca^2+^/PAAm/PNMA transparent conductive hydrogel had good mechanical properties, elastic properties, self-recovery and fatigue resistance.

### 3.3. Electro-Mechanical Properties and Strain-Sensing Performance of CMCS-Ca^2+^/PAAm/PNMA Transparent Conductive Hydrogels

**Figure 4:**
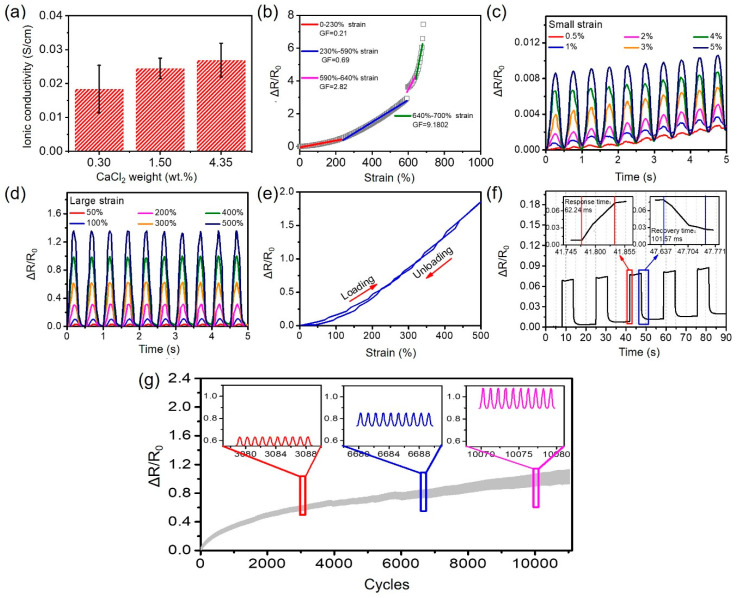
Electromechanical performances of CMCS-Ca^2+^/PAAm/PNMA hydrogels and their strain sensors. (**a**) Conductivity value as a function of different CaCl_2_ addition content; (**b**) relative resistance change and linear fit of the strain sensors made of CMCS-Ca^2+^/PAAm/PNMA hydrogels as a function of strain; (**c**,**d**) plots of ΔR/R_0_ vs. time with stretching–relaxation under different strains from 0.5% to 500%. (**e**) The ΔR/R_0_ curves of CMCS-Ca^2+^/PAAm/PNMA hydrogel under the 0% to 500% applied strain at the stretching rate of 100 mm/min. (**f**) Response and recovery time of the CMCS-Ca^2+^/PAAm/PNMA hydrogel sensors. (**g**) The resistance response of the CMCS-Ca^2+^/PAAm/PNMA hydrogel sensors over 11,000 stretching–relaxation cycles at 100% strain under 2000 mm/s tensile speed.

The presence of CaCl_2_ benefits the as-prepared CMCS-Ca^2+^/PAAm/PNMA hydrogels with good ionic conductivity without adding additional conductive agents. As the increase of the concentration of CaCl_2_, the conductivity of CMCS-Ca^2+^/PAAm/PNMA hydrogel increased (Figure 4a). When the concentration of CaCl_2_ was 4.35 wt.%, the conductivity of the hydrogel reached the maximum, which was 0.02688 S/cm. The relative resistance changing rates of the hydrogel changed significantly as the increasing of strain, indicating an excellent strain sensitivity (Figure 4b). Gauge factor (GF) is an important parameter to evaluate the sensitivity of strain sensors. According to the linear fitting results (Figure 4b), the strain response curve of CMCS-Ca^2+^/PAAm/PNMA conductive hydrogel can be divided into four regions, including 0–230%, 230–590%, 590–640%, 640–700%, and the GF of these four regions were 0.21, 0.69, 2.82, and 9.18, respectively [31,32]. Moreover, as shown in Figure 4c, the hydrogel can even detect a small deformation of 0.5%, showing excellent strain-sensing sensitivity. In addition, the responsiveness of the hydrogel under multiple strains was tested. Figure 4c,d showed that the hydrogel exhibited stable strain response in a wide strain range. Due to the double physical cross-linking network of CMCS-Ca^2+^/PAAm/PNMA hydrogel, the hydrogel showed a negligible response hysteresis at 500% strain (Figure 4e), which was one of the necessary properties of the strain sensor. Figure 4f showed that the hydrogel also exhibited faster strain response with response time and recovery time of 62.24 ms and 101.57 ms, respectively. In order to further explore the stability, the CMCS-Ca^2+^/PAAm/PNMA hydrogel was tested for >11,000 cycles under a strain of 100%, and the results are shown in Figure 4g. The relative resistance changing rate of the hydrogel showed excellent stability, which was of great significance for the long-term use of wearable sensors. Here, we compare our as-prepared conductive hydrogel with the reported works about the properties of transparency, fracture strain, tensile strength, toughness and conductivity (Table 1) [33,34,35,36,37,38].

**Figure 5:**
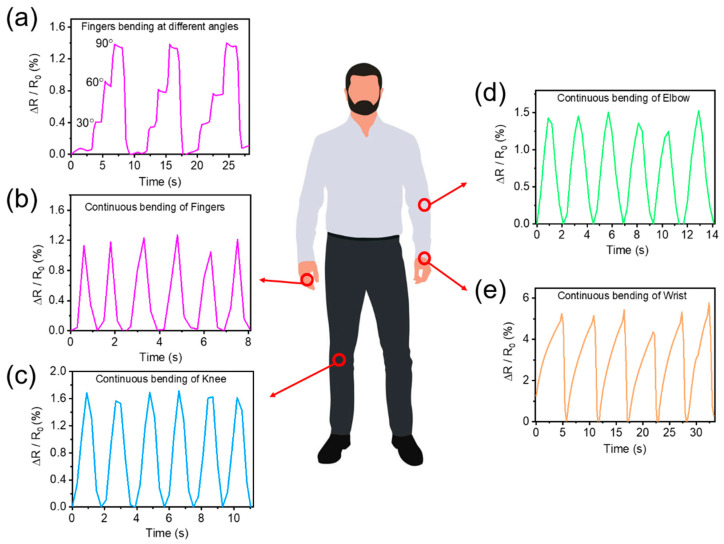
Body movement monitoring applications with the as-prepared CMCS-Ca^2+^/PAAm/PNMA hydrogels by attaching it to different parts of a human body. (**a**) and (**b**) fingers; (**c**) knees; (**d**) elbows; and (**e**) wrists.

Due to the excellent electro-mechanical properties of the CMCS-Ca^2+^/PAAm/PNMA conductive hydrogel, we used very high bond (VHB) tape to attach the hydrogel to the fingers to monitor joint movements. When the fingers were bent to different angles (from initial, 30 degree, 60 degree and 90 degree), the resistance change rate of the as-prepared hydrogels gradually increased and showed a step-like trend (Figure 5a). Moreover, when the fingers were repeatedly bent and straightened, the resistance change rate of the conductive hydrogel can also be monitored in real time (Figure 5b). In addition, we also adhered the CMCS-Ca^2+^/PAAm/PNMA conductive hydrogel to the knee (Figure 5c), elbow (Figure 5d) and wrist (Figure 5e) joints to monitor the movement of these joints and obtained the corresponding resistance change rate curve. It can be observed that the conductive hydrogel can obtain stable electrical signal output in different joint motions, which indicated that it can be used to monitor physiological signals and human movement.

## 4. Conclusions

In summary, we successfully prepared a CMCS-Ca^2+^/PAAm/PNMA transparent, tough, conductive hydrogel containing a bi-physical crosslinking network through in situ free radical polymerization. The CMCS-Ca^2+^/PAAm/PNMA conductive hydrogel had excellent light transmittance (>90%), high ionic conductivity, excellent tensile strength (2.65 MPa), good facture strain (707%), high elastic modulus (0.30 MPa), and excellent toughness (10.72 MJ/m^3^). In addition, due to the existence of the dual physical cross-linking network, the conductive hydrogel had good resilience, self-recovery and fatigue resistance. In addition, the conductive hydrogel had outstanding strain sensing performance with high sensitivity (maximum GF = 9.18, lowest detection limit was 0.5%), wide strain response range, faster response time and recovery time, negligible response hysteresis and excellent durability.

## Figures and Tables

**Table 1:** Brief summary of results reported on conductive hydrogels.

Hydrogels	Transparency	Fracture Strain	Tensile Strength	Toughness	Conductivity
	(%)	(%)	(MPa)	(MJ/m^3^)	(S/m)
PVA/cellulose nanofibrils/DMSO/H_2_O [33]	90%	660	2.1	5.25	3.2
PAAm/GE/Na_3_Cit [34]	non-transparent (milky white)	849	1.66	4.37	1.5
Cellulose/benzyltrimethyl ammonium hydroxide/epichlorohydrin [35]	non-transparent (black)	219	2	1.8	2.37
Fe^3+^/SL/PAA [36]	82% (red)	1680	0.052	0.59	7.0 × 10^−2^
PVA/GE/GL/NaCl [37]	Semi-transparent (N/A)	715	1.044	3.605	0.4
Adenosine monophosphate/quaternized chitosan/NaCl/PAAm [38]	92%	1731	0.347	2.8	1.45
This work	90%	707	2.65	10.72	2.688

PVA: polyvinyl alcohol; DMSO: dimethyl sulfoxide; GE: gelatin; SL: sulfonated lignin; PAA: polyacrylic acid; GL: glycerin.
